# Mechanistic Insights into Glycine’s Regulation of Milk Protein Synthesis via PI3K-AKT-mTOR Signaling

**DOI:** 10.3390/cimb48050453

**Published:** 2026-04-27

**Authors:** Xinyu Zhang, Yu Ding, Min Yang, Yuxin Zhou, Ruoshan Luo, Yang Yang, Hang Zhang, Wanping Ren, Liang Yang, Yong Wei, Yankun Zhao, Tongjun Guo, Wei Shao

**Affiliations:** 1 Xinjiang Key Laboratory of Meat and Milk Production Herbivore Nutrition, College of Animal Science, Xinjiang Agricultural University, Urumqi 830052, China; 18663955139@163.com (X.Z.); 18481902483@163.com (Y.D.); y1375724150@163.com (M.Y.); zyxgy1123@163.com (Y.Z.); 17809968072@163.com (R.L.); yy14799670731@163.com (Y.Y.); 17382543605@163.com (H.Z.); rwp15999154824@163.com (W.R.); yangliangagu@sina.com (L.Y.); wy-260@163.com (Y.W.); 2 Agricultural Products Quality and Safety Risk Assessment Laboratory of the Ministry of Agriculture and Rural Affairs (Urumqi) and the Xinjiang Agricultural Products Quality and Safety Laboratory, Xinjiang Academy of Agricultural Sciences, Institute of Agricultural Quality Standards and Testing Technology, Urumqi 830052, China; yankunzhao90@xaas.ac.cn; 3 Feed Research Institute, Xinjiang Uygur Autonomous Region Academy of Animal Husbandry Sciences, Urumqi 830052, China; guotaoxj@126.com

**Keywords:** glycine, MAC-T cells, α-casein, PI3K-AKT-mTOR signaling pathway

## Abstract

Amino acids play a dual role in milk protein synthesis, functioning as both metabolic precursors and signaling molecules. This study aimed to elucidate the molecular mechanism by which glycine regulates α-casein production in bovine mammary epithelial cells (MAC-T). Under serum-free conditions, MAC-T cells were exposed to graded concentrations of glycine (1.105, 2.209, 4.418, 8.836, 17.673, and 35.345 mM) for 24 h. α-Casein levels in cell lysates and culture supernatants were quantified by ELISA. Transcriptional activity of casein-encoding genes (*CSN1S1*, *CSN1S2*) and PI3K-AKT-mTOR pathway components was assessed by RT-qPCR. Phosphorylation status of pathway proteins was analyzed by Western blot. The functional involvement of the PI3K-AKT-mTOR pathway was validated using the specific inhibitor LY294002. Glycine stimulated α-casein synthesis and secretion in a concentration-dependent manner, with maximal efficacy at 4.418 mM. At this concentration, glycine upregulated *CSN1S1*, *CSN1S2*, and PI3K-AKT-mTOR pathway gene expression, and enhanced phosphorylation of the corresponding proteins. Inhibition of PI3K-AKT-mTOR signaling by LY294002 abolished glycine-induced α-casein synthesis, and this effect was reversed by glycine co-treatment. These findings demonstrate that glycine enhances α-casein synthesis through activation of the PI3K-AKT-mTOR pathway.

## 1. Introduction

Casein constitutes approximately 80% of total milk protein and serves as the pivotal component defining the nutritional and technological attributes of milk. As a premium source of animal-derived protein, milk’s nutritional value largely originates from its casein-dominated protein fraction [1]. Casein is primarily classified into three subtypes, among which α-casein constitutes approximately 40–50% of total milk protein [2]. The synthesis of milk proteins occurs primarily within bovine mammary epithelial cells [3]. These cells are capable of endogenously producing more than 90% of milk proteins by importing free amino acids from the circulation [4]. Thus, the supply of amino acids serves as the key determinant regulating the rate of milk protein biosynthesis. At the molecular level, amino acids possess a dual capacity in cellular physiology: they serve not only as essential precursors for protein assembly but also as intracellular signaling molecules that modulate key pathways and influence the transcription of milk protein-related genes [5]. In bovine mammary epithelial cells, essential amino acids such as lysine and leucine promote cell proliferation and casein synthesis by activating the mTOR signaling pathway [6,7]. Similarly, the non-essential amino acid glutamine regulates casein synthesis through the mTOR pathway [8]. Further research indicates that the regulatory effects of these amino acids depend on the PI3K-AKT signaling axis upstream of mTOR. For instance, methionine and leucine activate the PI3K-AKT-mTOR cascade to promote casein synthesis and cell proliferation, and this effect is completely blocked by the PI3K-specific inhibitor LY294002 [9,10]. Glycine, as a conditionally essential amino acid for young mammals, is not only involved in protein synthesis but also plays a key role in one-carbon metabolism and glutathione synthesis [11]. These properties suggest that it may have a signaling function. A previous study has shown that low concentrations of glycine can promote the proliferation of bovine mammary epithelial cells [12]. The number of bovine mammary epithelial cells is one of the key factors affecting the synthesis of milk proteins [13], and this provides a favorable cellular basis for casein synthesis. Moreover, glycine significantly activates the transport of branched-chain amino acids, indirectly promoting casein synthesis in bovine mammary epithelial cells [14]. However, none of these studies directly addressed whether glycine can regulate casein synthesis through specific signaling pathways. Based on this, we hypothesize that glycine may regulate α-casein synthesis in bovine mammary epithelial cells by influencing the PI3K-AKT-mTOR signaling pathway. To verify this hypothesis, this study used MAC-T cells as an in vitro model to investigate the effects of glycine on the synthesis and secretion of α-casein, the expression of α-casein-encoding genes, and the activity of the PI3K-AKT-mTOR signaling pathway, thereby clarifying the mechanism by which glycine promotes α-casein synthesis in bovine mammary epithelial cells.

## 2. Materials and Methods

### 2.1. Materials

In this study, cell culture was performed using Dulbecco’s Modified Eagle medium (DMEM, C11965500BT, Thermo Scientific, Waltham, MA, USA) as the basal medium. The medium was supplemented with 10% fetal bovine serum (FBS, 10099-141C, Thermo Scientific, Waltham, MA, USA) and 0.25% trypsin-EDTA (25200-056, Thermo Scientific, Waltham, MA, USA) for routine cell passage. Glycine (G8790, Sigma-Aldrich, St. Louis, MO, USA) and the PI3K inhibitor LY294002 (HY-10108, MedChemExpress, Monmouth Junction, NJ, USA) were used as key supplements for the experimental treatments.

### 2.2. Cell Culture

The bovine mammary epithelial cell line MAC-T (BFN607200656, Qingqi Biotechnology Development Co., Ltd., Shanghai, China) was purchased and used for all in vitro experiments. These cells exhibited typical epithelial cobblestone morphology, maintained stable proliferation over 30 passages, and retained the capacity for casein synthesis. Cells were routinely cultured in high-glucose DMEM supplemented with 10% FBS and maintained at 37 °C in a humidified atmosphere containing 5% CO_2_.

For experimental treatments, cells were subjected to a 12 h serum starvation period, followed by 24 h exposure to glycine at specified concentrations: 1.105, 2.209, 4.418, 8.836, 17.673, and 35.345 mM (corresponding to 1×, 2×, 4×, 8×, 16×, and 32×, where 1× = 1.105 mM). The positive control (PC) group consisted of cells maintained in complete medium (DMEM with 10% FBS) without serum starvation or glycine treatment, serving as a reference for normal cell growth and casein synthesis capacity. The negative control (0× Gly) group underwent the same 12 h serum starvation followed by 24 h culture in serum-free medium without glycine supplementation.

To validate the involvement of the PI3K-AKT-mTOR pathway, we first determined the appropriate concentration of the specific inhibitor LY294002. Cells were cultured in complete medium (10% FBS) with LY294002 at 10, 25, or 50 μM for 24 h. Based on the most pronounced PI3K phosphorylation inhibition without detectable cytotoxicity, 50 μM was selected for subsequent experiments. Cells were then divided into four groups under serum-starvation conditions: negative control (no supplement), LY294002 alone (50 μM), optimal glycine alone (the concentration with the highest α-casein level in the dose–response experiment), and optimal glycine plus LY294002 (50 μM). After 24 h of treatment, cells were harvested for Western blot analysis of α-casein and PI3K-AKT-mTOR pathway phosphorylation.

### 2.3. ELISA

The concentration of α-casein was determined by ELISA kit (JONLNBIO, JL22469, Shanghai, China) according to the manufacturer’s instructions and a previously established method [15]. To measure intracellular α-casein, cells were lysed by ultrasonication (ThermoFisher, FB50, Waltham, MA, USA). To measure extracellular (secreted) α-casein, cell culture supernatants were collected after the 24 h treatment period. Both cell lysates and culture supernatants were then subjected to ELISA following the kit protocol.

### 2.4. RT-qPCR

Total RNA was extracted using TRIzol™ Reagent (Invitrogen, 15596026CN, Carlsbad, CA, USA). cDNA was synthesized from total RNA using the PrimeScript™ RT Kit (TaKaRa, RR037A, Shiga, Japan) on a gradient thermal cycler. Real-time PCR amplifications were performed on a CFX Connect Real-Time PCR Detection System (BioRad, 17005940, Hercules, CA, USA) with TB Green Premix Ex Taq™ (TaKaRa, RR420A, Shiga, Japan) to quantify transcripts associated with α-casein synthesis and the PI3K-AKT-mTOR signaling pathway. β-actin was used as the endogenous control, and relative gene expression levels were calculated using the 2^−ΔΔCt^ method. The primers used in this study are listed in Table 1.

### 2.5. Western Blot

Samples for Western blotting were prepared in parallel with those for RT-qPCR under identical cell culture conditions. Following two washes with ice-cold PBS, cells were then lysed directly in their culture wells. Lysis was performed with 100 μL of ice-cold RIPA buffer (ASPEN, AS1004, Wuhan, China) that contained added protease (Roche, 04693159001, China) and phosphatase inhibitors (ASPEN, AS1008, Wuhan, China). Lysis was carried out for 5 min. The cells and reagents were scraped from the wells using a cell scraper and transferred into a 1.5 mL centrifuge tube, which was incubated on ice for 30 min. During this period, the cells were resuspended by repeated pipetting to ensure complete cell lysis. To obtain the supernatant, samples were centrifuged at 12,000× *g* for 5 min at 4 °C. To ensure consistent loading for subsequent analysis, total protein concentrations in the collected supernatants were measured using the BCA Protein Assay Kit (ASPEN) and equalized. For loading, an aliquot of 40 μg protein from each normalized sample was combined with loading buffer at a 4:1 (sample:buffer) ratio, followed by denaturation at 100 °C for 5 min. Subsequently, proteins were separated by SDS-PAGE and then transferred onto a PVDF membrane (Millipore, IPVH00010, Burlington, MA, USA). The transferred PVDF membrane was blocked in blocking solution (Blt-PO Buffer, ASPEN, AS1033, Wuhan, China) for 1 h at room temperature, and then incubated with the primary antibody solution. After three washes with TBST (prepared from TBS, ASPEN, AS1024, China and Tween-20, ASPEN, AS1100, Wuhan, China), the membrane was incubated with the secondary antibody for 30 min at room temperature. The membrane was then subjected to four consecutive 5 min washes with TBST at room temperature under gentle agitation. The membrane was transferred to a cassette, incubated with ECL chemiluminescent substrate (ASPEN, AS1059, Wuhan, China), and then exposed in the darkroom. Blots were digitized for archiving, and the intensities of target bands were quantified by densitometry using AlphaEase FC 4.0.0 software. Comprehensive details regarding these antibodies are provided in Table 2.

### 2.6. Statistical Analysis

Data management and preliminary processing were conducted in Excel 2016. Subsequently, statistical analysis was carried out using SPSS 19.0, employing one-way ANOVA with Duncan’s multiple range test for post hoc comparisons. For data presentation, results are expressed as the mean ± SEM. Statistical significance was defined as *p* < 0.05, and high significance as *p* < 0.01.

## 3. Results

### 3.1. Dose-Dependent Stimulation of α-Casein Synthesis and Secretion by Glycine

**Figure 1:**
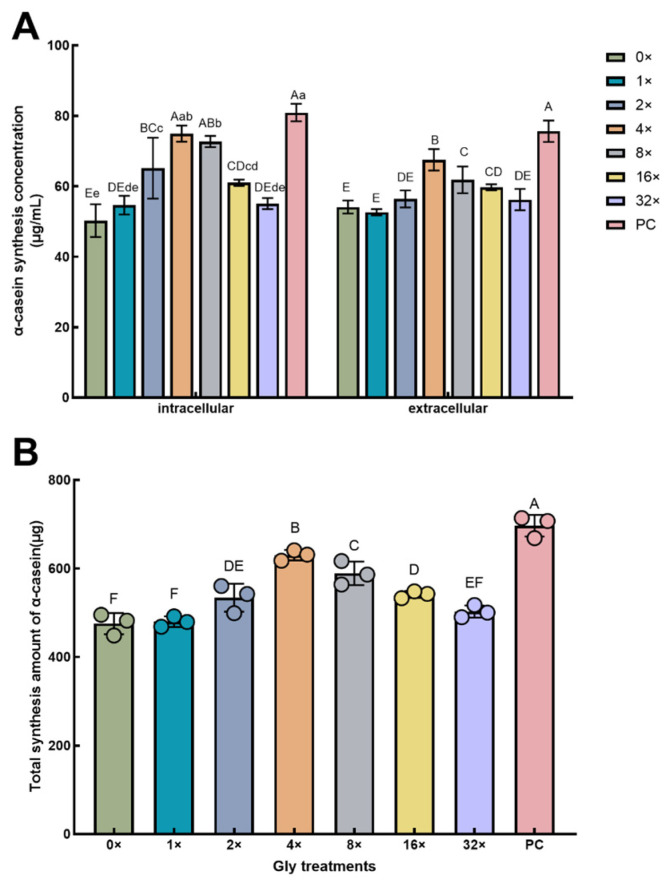
Dose-Dependent Stimulation of α-Casein Synthesis and Secretion by Glycine. (**A**) Intracellular and extracellular α-casein concentrations. (**B**) Total α-casein production. All values represent the mean ± SEM (*n* = 3). In the graphical representation, statistically significant differences (*p* < 0.05, Duncan’s test) are denoted by different lowercase letters, whereas distinct uppercase letters indicate highly significant differences (*p* < 0.01). This convention for data presentation and statistical annotation applies to all subsequent figures unless otherwise stated.

Glycine supplementation at 2×, 4×, 8×, and 16× concentrations significantly elevated both intracellular α-casein levels and total α-casein production in MAC-T cells relative to the 0× Gly group (*p* < 0.01, Figure 1). Compared with the 0× Gly group, glycine supplementation at 4× Gly, 8× Gly, and 16× Gly significantly increased extracellular α-casein secretion in MAC-T cells (*p* < 0.01, Figure 1A). Compared with PC group, glycine supplementation at 0× Gly, 1× Gly, 2× Gly, 16× Gly, and 32× Gly significantly reduced intracellular α-casein synthesis (*p* < 0.01, Figure 1B). Moreover, all glycine supplementation groups exhibited significantly decreased levels of extracellular α-casein secretion and total α-casein synthesis (*p* < 0.01, Figure 1A,B). When the glycine supplementation level was 8× Gly, intracellular α-casein synthesis was significantly decreased (*p* < 0.05, Figure 1B).

### 3.2. Transcriptional Responses of α-Casein Genes to Glycine

**Figure 2:**
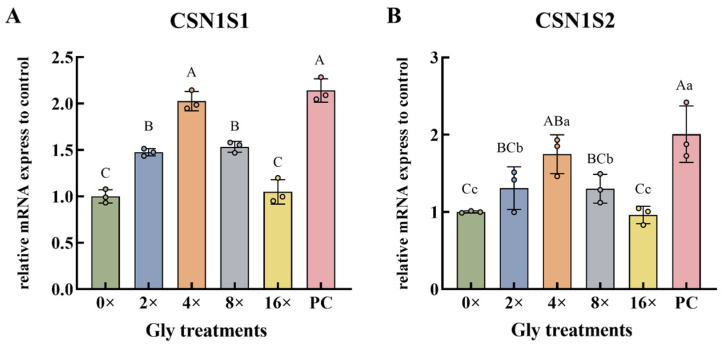
Transcriptional Responses of α-Casein Genes to Glycine. The relative expression levels of *CSN1S1* (**A**) and *CSN1S2* (**B**) genes (*n* = 3), respectively. In the graphical representation, statistically significant differences (*p* < 0.05, Duncan’s test) are denoted by different lowercase letters, whereas distinct uppercase letters indicate highly significant differences (*p* < 0.01). Statistical significance and annotation conventions are as described in the Figure 1 legend.

Glycine treatment at 2×, 4×, and 8× concentrations induced a significant up-regulation in *CSN1S1* gene expression compared to the 0× Gly control (*p* < 0.01, Figure 2A). In contrast, significant up-regulation of *CSN1S2* was observed only at the 4× Gly supplementation level (*p* < 0.01, Figure 2B). Compared with the 0× Gly control, *CSN1S2* transcript levels were significantly increased at both 2× and 8× Gly groups (*p* < 0.05, Figure 2B). At the 4× Gly concentration, *CSN1S1* expression reached its maximum level, significantly higher than that in the 0×, 2×, 8×, and 16× Gly groups (*p* < 0.01, Figure 2A). At the same concentration, *CSN1S2* expression was significantly greater than in the 0× and 16× groups (*p* < 0.01) and also higher than in the 2× and 8× Gly groups (*p* < 0.05, Figure 2B). A significant down-regulation of both *CSN1S1* and *CSN1S2* was evident in the 0×, 2×, 8×, and 16× Gly groups relative to the PC group (*p* < 0.01, Figure 2A,B).

### 3.3. Glycine Activates Gene Expression of PI3K-AKT-mTOR Upstream Components

**Figure 3:**
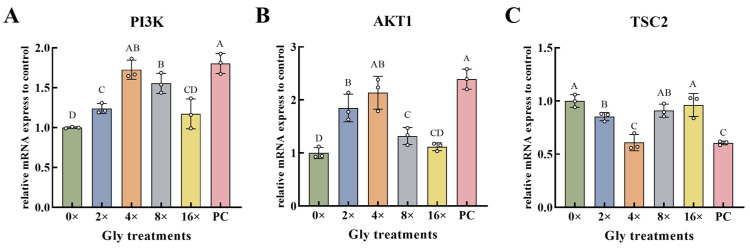
Glycine Activates Gene Expression of PI3K-AKT-mTOR Upstream Components. (**A**–**C**) The relative expression levels of the *PI3K* (**A**), *AKT1* (**B**), and *TSC2* (**C**) genes (*n* = 3), respectively. In the graphical representation, statistically significant differences (*p* < 0.05, Duncan’s test) are denoted by different lowercase letters, whereas distinct uppercase letters indicate highly significant differences (*p* < 0.01). Statistical significance and annotation conventions are as described in the Figure 1 legend.

Compared with the 0× Gly control, *PI3K* and *AKT1* expression was significantly up-regulated at 2×, 4×, and 8× Gly (*p* < 0.01, Figure 3A,B). In contrast, *TSC2* transcript levels were significantly reduced at 2× and 4× Gly (*p* < 0.01, Figure 3C). At the 4× Gly concentration, *PI3K* and *AKT1* expression levels were highest, and *TSC2* expression was lowest among all groups. At this concentration, *PI3K* abundance reached its peak and was significantly higher than in the 0×, 2×, and 16× Gly groups (*p* < 0.01, Figure 3A). *AKT1* levels exhibited a similar pattern, showing significantly greater expression compared to the 0×, 8×, and 16× Gly groups (*p* < 0.01, Figure 3B). In contrast, *TSC2* expression at 4× Gly was significantly downregulated relative to all other tested groups (*p* < 0.01, Figure 3C). At the specified glycine concentrations (0×, 2×, 8×, and 16× Gly), transcript levels of *PI3K* and *AKT1* were suppressed relative to the PC group, whereas *TSC2* expression was elevated (*p* < 0.01, Figure 3A–C).

### 3.4. Induction of Downstream mTOR Effector Gene Transcription by Glycine

**Figure 4:**
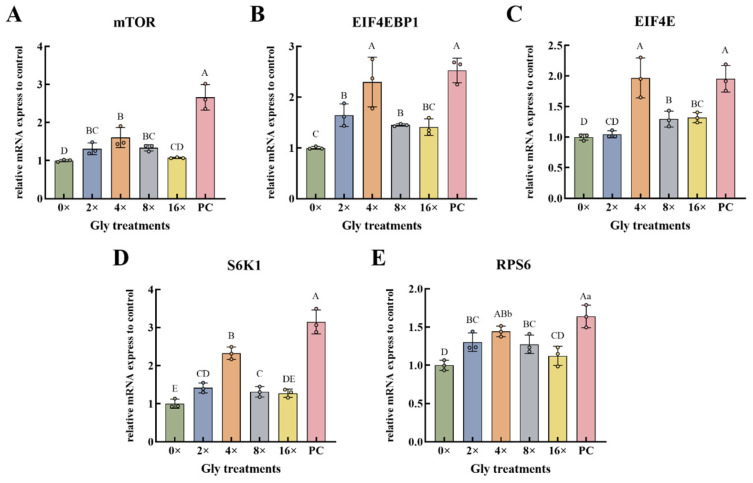
Induction of Downstream mTOR Effector Gene Transcription by Glycine. (**A**–**E**) The relative expression levels of *mTOR* (**A**), *EIF4EBP1* (**B**), *EIF4E* (**C**), *S6K1* (**D**), and *RPS6* (**E**) genes (*n* = 3), respectively. In the graphical representation, statistically significant differences (*p* < 0.05, Duncan’s test) are denoted by different lowercase letters, whereas distinct uppercase letters indicate highly significant differences (*p* < 0.01). Statistical significance and annotation conventions are as described in the Figure 1 legend.

Relative to the 0× Gly control, the expression of *mTOR*, *EIF4EBP1*, *S6K1*, and *RPS6* was elevated in the 2×, 4×, and 8× Gly groups, while *EIF4E* demonstrated increased expression at 4×, 8×, and 16× Gly (*p* < 0.01, Figure 4A–E). Maximal transcript levels for all five genes occurred at 4× Gly. At this concentration, *EIF4EBP1*, *EIF4E*, and *S6K1* transcripts were significantly elevated, exceeding levels in both the control group and all other glycine-treated groups (*p* < 0.01, Figure 4B–D). In comparison with the PC group, *mTOR* and *S6K1* exhibited significant suppression of expression at each of the glycine concentrations examined (*p* < 0.01, Figure 4A,D). *EIF4EBP1*, *EIF4E*, and *RPS6* were also downregulated at most concentrations (0×, 2×, 8×, and 16× Gly, *p* < 0.01), with the exception of RPS6 at 4× Gly, which showed a smaller magnitude of decrease that remained significant (*p* < 0.05, Figure 4A,D,E).

### 3.5. Glycine Triggers Phosphorylation of PI3K, AKT, and TSC2 Proteins

**Figure 5:**
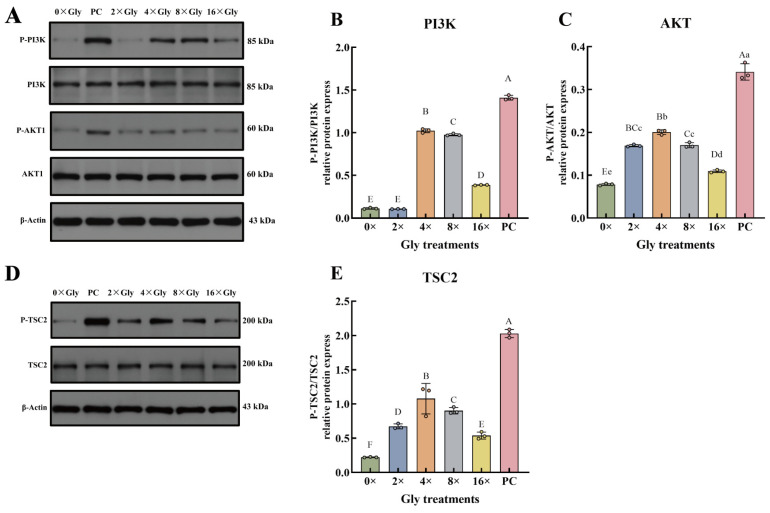
Glycine Triggers Phosphorylation of PI3K, AKT1, and TSC2 Proteins. (**A**,**D**) The phosphorylation of PI3K, AKT1, and TSC2 was analyzed by Western blot using phospho-specific and total protein antibodies. (**B**,**C**,**E**) Normalization was performed to the respective total protein and to β-actin, and the P-protein/total protein ratios were calculated. Data are presented as mean ± SEM (*n* = 3). In the graphical representation, statistically significant differences (*p* < 0.05, Duncan’s test) are denoted by different lowercase letters, whereas distinct uppercase letters indicate highly significant differences (*p* < 0.01). Statistical significance and annotation conventions are as described in the Figure 1 legend.

Phosphorylation of PI3K was up-regulated at 4×, 8×, and 16× Gly, while phosphorylation of AKT1 and TSC2 increased at 2×, 4×, 8×, and 16× Gly compared with the 0× Gly control (*p* < 0.01, Figure 5A–E). At the 4× Gly concentration, phosphorylation levels of all three proteins reached their peak. PI3K phosphorylation at this concentration was significantly higher than in all other groups (*p* < 0.01, Figure 5B). AKT1 phosphorylation exceeded that in the 0×, 8×, and 16× Gly groups (*p* < 0.01, Figure 5C) and was also elevated relative to the 2× Gly group (*p* < 0.05, Figure 5C). TSC2 phosphorylation at 4× Gly was maximal, with a significant increase compared to all other treatment groups (*p* < 0.01, Figure 5E). Phosphorylation of the upstream proteins PI3K, AKT1, and TSC2 was markedly lower across all glycine supplementation groups when compared to the PC group (*p* < 0.01, Figure 5A–E).

### 3.6. Enhanced Phosphorylation of mTOR, 4EBP1, S6K1, and RPS6 by Glycine

**Figure 6:**
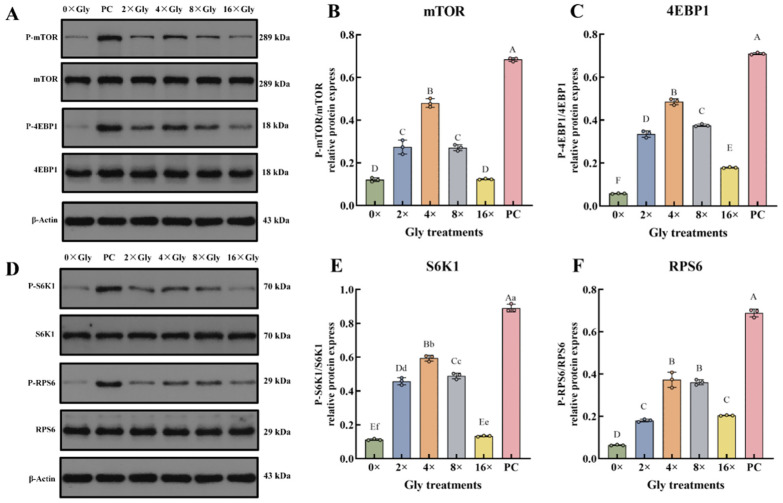
Enhanced Phosphorylation of mTOR, 4EBP1, S6K1, and RPS6 by Glycine. (**A**,**D**) The phosphorylation of mTOR, 4EBP1, S6K1, and RPS6 was analyzed by Western blot using phospho-specific and total protein antibodies. (**B**,**C**,**E**,**F**), Normalization was performed to the respective total protein and to β-actin, and the P-protein/total protein ratios were calculated. Data are presented as mean ± SEM (*n* = 3). In the graphical representation, statistically significant differences (*p* < 0.05, Duncan’s test) are denoted by different lowercase letters, whereas distinct uppercase letters indicate highly significant differences (*p* < 0.01). Statistical significance and annotation conventions are as described in the Figure 1 legend.

Compared with the 0× Gly control, phosphorylation of 4EBP1 and RPS6 was enhanced over a broad concentration range (2× to 16× Gly), whereas phosphorylation of mTOR and S6K1 increased specifically at 2×, 4×, and 8× Gly (*p* < 0.01, Figure 6A,E). S6K1 also exhibited a significant increase at 16× Gly (*p* < 0.05, Figure 6F). For all four proteins, phosphorylation reached its peak at 4× Gly. 4EBP1 and RPS6 phosphorylation showed the highest levels at this concentration, significantly exceeding those in the control and all other treatment groups (*p* < 0.01, Figure 6C,F). Similarly, phosphorylated mTOR and S6K1 levels at 4× Gly were significantly greater than in the control and all other treatment groups (*p* < 0.01, Figure 6B,E). A significant reduction in the phosphorylation of mTOR, 4EBP1, S6K1, and RPS6 was observed in all glycine-treated groups relative to the PC control (*p* < 0.01, Figure 6B,C,E,F).

### 3.7. PI3K Inhibitor LY294002 Abrogates Glycine-Mediated Effects, Validating Pathway Specificity

**Figure 7:**
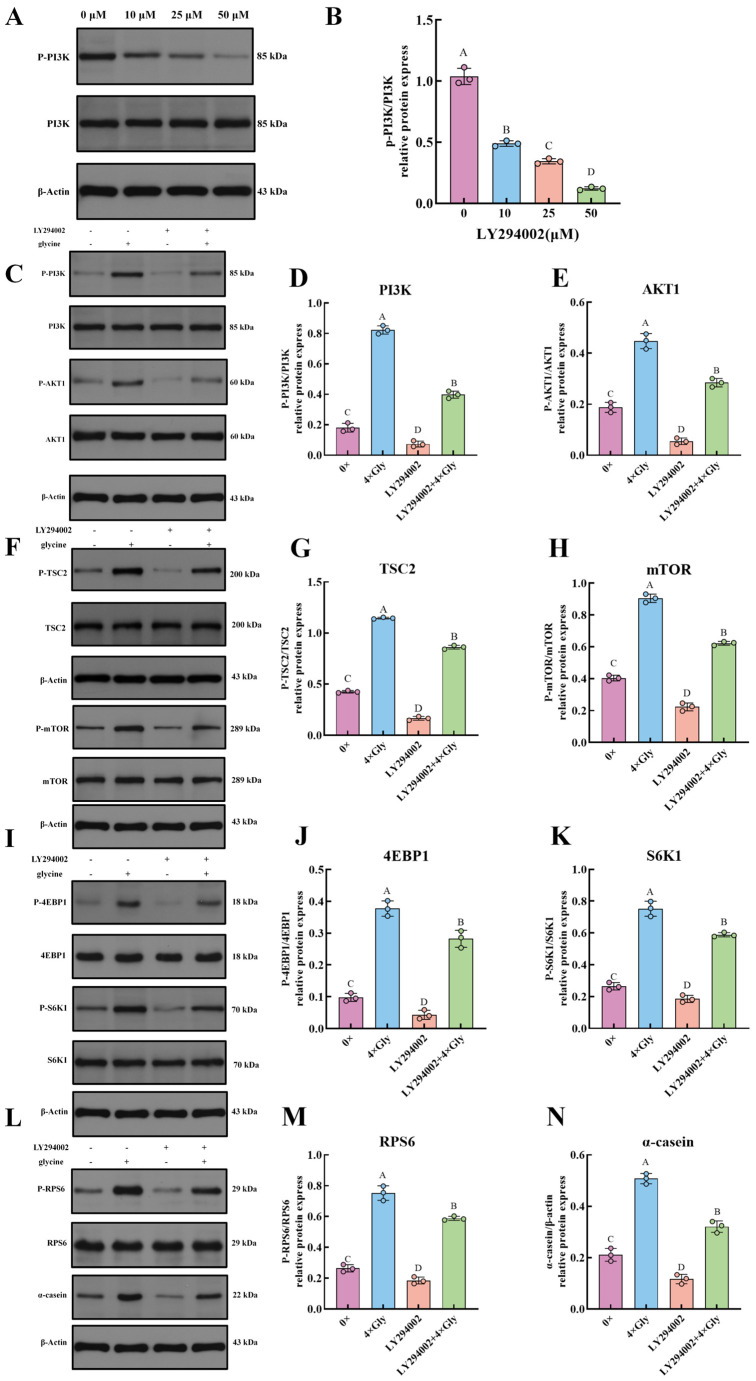
PI3K Inhibitor LY294002 Abrogates Glycine-Mediated Effects, Validating Pathway Specificity. (**A**,**C**,**F**,**I**,**L**) Western blot analysis was performed using antibodies against the phosphorylated (P-) and total forms of PI3K, AKT1, TSC2, mTOR, 4EBP1, S6K1, and RPS6, as well as against α-casein. (**B**,**D**,**E**,**G**,**H**,**J**,**K**,**M**,**N**) The ratios of P-protein to total protein were calculated following quantification of band intensities, using β-actin as an internal control. Data are presented as mean ± SEM (*n* = 3). In the graphical representation, statistically significant differences (*p* < 0.05, Duncan’s test) are denoted by different lowercase letters, whereas distinct uppercase letters indicate highly significant differences (*p* < 0.01). Statistical significance and annotation conventions are as described in the Figure 1 legend.

Compared with the 0× Gly group, LY294002 at 10, 25, and 50 μM significantly inhibited PI3K phosphorylation (*p* < 0.01, Figure 7B). The maximal suppression was observed at 50 μM, a concentration that exerted a significantly stronger inhibitory effect than either 10 or 25 μM (*p* < 0.01, Figure 7B), and was therefore selected for subsequent experiments. Treatment with 50 μM LY294002 robustly suppressed phosphorylation of PI3K, AKT1, TSC2, mTOR, 4EBP1, S6K1, and RPS6, concomitant with reduced α-casein synthesis, compared with the 0× Gly control (*p* < 0.01, Figure 7C–N). In the presence of both LY294002 and glycine, phosphorylation levels of these proteins and α-casein prodution were significantly restored relative to the LY294002-only group (*p* < 0.01, Figure 7C–N).

## 4. Discussion

### 4.1. Dose-Dependent Stimulation of α-Casein Synthesis and Secretion by Glycine

Amino acid levels significantly affect the synthetic function of mammary epithelial cells in dairy cows. Conejos et al. [16] demonstrated that β-casein synthesis in bovine mammary epithelial cells was highest when L-arginine was supplemented at 0.9 mM. In the present study, glycine promoted α-casein synthesis and secretion in MAC-T cells in a concentration-dependent manner, with the optimal effect at 4×. 2× glycine significantly increased intracellular α-casein levels but did not significantly affect extracellular secretion, whereas 4× and higher concentrations markedly promoted α-casein secretion. This suggests that low glycine levels may satisfy basal synthetic requirements but are insufficient to fully activate the secretory pathway. Amino acids regulate secretion by modulating protein synthesis and transport pathways [17]. Amino acid deficiency inhibits protein transport in the secretory pathway [18]. Thus, higher glycine concentrations likely provide additional substrates and energy to support the loading capacity of secretory organelles, such as the endoplasmic reticulum, thereby ensuring efficient processing and release of synthesized α-casein. The increase in total α-casein synthesis resulted from the combined improvement of both synthesis and secretion, and its trend was consistent with intracellular α-casein levels, indicating that enhanced synthetic capacity was the main driver of increased total yield. When glycine was added at 1×, α-casein synthesis did not change significantly, suggesting that limited glycine availability restricts amino acid utilization efficiency, leading to reduced synthesis and secretion. As the glycine concentration continued to rise, α-casein levels showed a downward trend. Studies have shown that when essential amino acid supply exceeds the metabolic demand of dairy cow mammary epithelial cells, further increasing the supply reduces utilization efficiency rather than promoting casein synthesis [19]. Therefore, excessively high glycine concentrations may disrupt cellular amino acid balance, leading to relative insufficiency in the transport or utilization of other essential amino acids (lysine and methionine), thereby limiting casein synthesis. This interpretation is consistent with the findings of M Sun et al. [20], who reported that increasing arginine concentration in BMEC culture medium significantly decreased the utilization of other amino acids and α-casein synthesis.

### 4.2. Transcriptional Responses of α-Casein Genes to Glycine

Amino acids serve not only as the precursors of milk protein synthesis, but also as signaling molecules that regulate this process. By activating specific signaling pathways, amino acids modulate the promoter activity and mRNA abundance of casein-encoding genes such as *CSN1S1* and *CSN1S2*, thereby promoting casein synthesis. When the ratio of lysine to methionine was 3:1, the expression levels of casein coding genes (*CSN1S1*, *CSN1S2*, *CSN2*, *CSN3*) in BMEC cells were significantly upregulated, and the concentration of casein synthesized was increased [21]. In the present study, glycine upregulated both *CSN1S1* and *CSN1S2* expression in a concentration-dependent manner. However, *CSN1S2* responded only at a narrower range of glycine concentrations compared with *CSN1S1*, suggesting gene-specific sensitivity to glycine signaling. This differential regulation may reflect distinct promoter architectures or transcription factor requirements for each casein gene. The optimal effect on both genes was observed at 4× glycine, which aligns with the concentration that maximized α-casein protein synthesis.

### 4.3. Molecular Mechanisms Underlying Glycine-Induced α-Casein Synthesis via PI3K-AKT-mTOR Activation

Amino acids serve as both metabolic substrates and signaling molecules. Through Rag GTPases, they promote mTORC1 recruitment to the lysosomal membrane, leading to its activation [22]. The PI3K-AKT-mTOR cascade integrates diverse extracellular signals to regulate key cellular processes [23]. PI3K activation generates PIP3, which recruits and activates AKT. AKT then phosphorylates TSC2, inhibiting the TSC1/TSC2 complex and thereby derepressing Rag GTPase, a critical step for mTOR activation [24]. This pathway is a known driver of milk protein synthesis in MAC-T cells. For example, prolactin activates PI3K-AKT-mTOR, upregulates LAT1, and increases amino acid import and casein output [25]. Similarly, leucine promotes PI3K phosphorylation and subsequent AKT/mTORC1 activation, enhancing translation via S6K1 [26]. In the present study, glycine activated the PI3K-AKT axis at both transcriptional and post-translational levels. The expression of PI3K and AKT1, as well as their phosphorylation, increased in a concentration-dependent manner, peaking at the optimal glycine concentration (4×). TSC2 phosphorylation followed a similar pattern, consistent with upstream PI3K-AKT activation. Notably, TSC2 mRNA levels were reduced at lower glycine concentrations, suggesting a negative feedback loop whereby activated mTOR signaling suppresses TSC2 expression to reinforce its own activity. This feedback, together with the downregulation of TSC2 transcription, further relieves inhibition of the mTOR pathway and ensures full activation of downstream effectors.

When mTOR is activated, it phosphorylates its downstream targets 4EBP1 and S6K1 to activate them [27]. 4EBP1 inhibits protein translation by binding to eIF4E, and mTOR phosphorylates 4EBP1 to release eIF4E, thereby promoting the formation of eIF4F complex and initiating 5′cap-dependent mRNA translation [28]. In turn, activated S6K1 phosphorylates RPS6, thereby enhancing ribosome biogenesis and translation efficiency and ultimately promoting protein synthesis [29]. In our experiments, glycine upregulated the mRNA levels of mTOR, EIF4EBP1, S6K1, and RPS6, as well as their phosphorylation, in a concentration-dependent manner. All responses peaked at the optimal glycine concentration and declined at higher concentrations, demonstrating a biphasic dose–response pattern. These data indicate that glycine modulates both the transcriptional and post-translational activation of the mTOR axis in a concentration-dependent fashion. Within bovine mammary epithelial cells, a homeostatic ratio of amino acids serves as a key coordinator that enables mTOR pathway activation and subsequently drives milk protein production [30]. By modulating the intracellular amino acid balance, glycine at appropriate concentrations effectively activates the mTOR signaling pathway. This activation culminates in the peak induction of gene expression and protein phosphorylation, ultimately driving enhanced protein synthesis.

It should be noted that the pathway activity in the positive control (PC) group, cultured with serum, was higher than in any glycine-treated group. This is expected because serum contains a broad spectrum of mitogens and growth factors, such as IGF and EGF, that act through receptor tyrosine kinases to activate PI3K-AKT-mTOR and other pathways including MAPK [31,32,33,34,35]. The multifactorial stimulation provided by serum contrasts with the single-amino-acid stimulus of glycine under serum-free conditions, which lacks synergistic multi-target effects [36]. Thus, our data highlight that glycine acts as a specific signaling molecule, but its effect is additive rather than a full substitute for the complex nutritional environment. Future studies should explore the synergistic actions of glycine with other nutrients.

### 4.4. Functional Verification of the PI3K-AKT-mTOR Pathway in Glycine-Induced α-Casein Synthesis

Studies have shown that glycine activates the AKT-mTOR signaling pathway in porcine small intestinal epithelial cells, thereby promoting S6K1 and 4EBP1 phosphorylation and enhancing protein synthesis efficiency [37]. In MAC-T cells, treatment with LY294002 suppressed the phosphorylation of key signaling molecules including PI3K, AKT1, mTOR, 4EBP1, S6K1, and RPS6, and concurrently inhibited α-casein synthesis. As a specific PI3K inhibitor, LY294002 competitively binds to the ATP-binding site of PI3K, blocking its catalytic activity and thereby inhibiting AKT1 activation [38,39]. Blockade of the upstream signal thus indirectly suppresses phosphorylation of mTOR and its downstream targets, reducing α-casein synthesis [40]. Our inhibitor assay established a functional requirement for PI3K-AKT-mTOR signaling in α-casein synthesis. Glycine alleviated the LY294002-induced blockade, restoring phosphorylation cascades and α-casein production. These findings confirm that glycine acts specifically through this pathway, consistent with prior work in mouse myoblasts [41] and hepatocytes [42] showing that glycine stimulates protein synthesis. Thus, in mammary cells, glycine promotes α-casein production not merely as a metabolic substrate but also as a signaling activator of the PI3K-AKT-mTOR pathway.

## 5. Conclusions

This study demonstrates that glycine promotes α-casein synthesis in MAC-T cells through activation of the PI3K-AKT-mTOR signaling pathway. The optimal effect was observed at 4× glycine (4.418 mM). Inhibitor experiments confirmed that this pathway is functionally required for glycine’s effect. These findings establish glycine as a signaling molecule in mammary nutrient sensing and provide a mechanistic framework for amino acid regulation of milk protein synthesis.

## Figures and Tables

**Table 1:** Primer sequence information.

Genes	Accession No.	Primer Sequences (5′→3′)
*PI3K*	XM_024988896	F:ATGGTGATGATTTACGGCAGGATATG R:TAAGGTAGCATCCGAAGGTCCAG
*AKT1*	XM_024981592	F:ATCATGCAGCACCGATTCTT R:AAATACCTGGTGTCCGTCTCA
*TSC2*	XM_059881502	F:GAGACACATCACCTACTTGGAAGAAG R:ACTAAGTTCACGAGCACCAGGAG
*mTOR*	XM_001788228.1	F:ATGCTGTCCCTGGTCCTTATG R:GGGTCAGAGAGTGGCCTTCAA
*EIF4EBP1*	NM_001077893.2	F:GAACTCACCTGTGACCAAGA R:CTCAAACTGTGACTCTTCACC
*EIF4E*	NM_174310.1	F:ACGAAGTGACCTCGATCGTT R:AGTAGCTGTGTCTGCATGGG
*S6K1*	NM_205816.1	F:CTGGGGAAGAGGTGCTTCAG R:GTGCTCTGGTCGTTTGGAGA
*RPS6*	NM_001010.2	F:AAGAGCTAGCAGAATCCGCA R:CGTGGAGTAACAAGACGCTG
*CSN1S1*	NM_181029.2	F:TCAACCCAGCTTGCTGCTTCTTCC R:GCCTAGCAAGAGCAACAGCCACAA
*CSN1S2*	NM_174528.2	F:AGCAGCTCTCCACCAGTGAGGAAA R:TGGGGCAAGGCGAATTTCTGGT
*β-actin*	NM_173979.3	F:GTCATCACCATCGGCAATGAG R:AATGCCGCAGGATTCCATG

**Table 2:** Antibody information.

	Antibody	Source	Item Number	Dilution Methods	Dilution Ratio
Primary antibody	β-Actin	tdybio	TDY051	5% egg white	1:10,000
	p-PI3K	affbiotech	AF4372	5% egg white	1:1000
	PI3K	Proteintech	60225-1-Ig	5% egg white	1:2000
	p-AKT1	Proteintech	66444-1-Ig	5% egg white	1:500
	AKT1	Proteintech	60203-2-Ig	5% egg white	1:1000
	p-TSC2	bioss	bs-3442R	5% egg white	1:500
	TSC2	affbiotech	AF6334	5% egg white	1:1000
	p-mTOR	bioss	bs-3494R	5% egg white	1:500
	mTOR	bioss	bs-1992R	5% egg white	1:1000
	p-4EBP1	affbiotech	AF3432	5% egg white	1:1000
	4EBP1	affbiotech	AF6432	5% egg white	1:2000
	p-S6K1	affbiotech	AF3228	5% egg white	1:1000
	S6K1	affbiotech	AF6226	5% egg white	1:2000
	p-RPS6	affbiotech	AF3354	5% egg white	1:500
	RPS6	biorbyt	orb585017	5% egg white	1:1000
Secondary antibody	HRP-Goat anti Rabbit	ASPEN	AS1107	5% skim milk	1:10,000
	HRP-Goat anti Mouse	ASPEN	AS1106	5% skim milk	1:10,000
	HRP-Rabbit anti Goat	ASPEN	AS1108	5% skim milk	1:10,000
	HRP-Goat anti Rat	ASPEN	AS1093	5% skim milk	1:10,000

## Data Availability

The original contributions presented in this study are included in the article. Further inquiries can be directed to the corresponding author.
